# Assessing heavy metal accumulation in the invasive blue crab (*Callinectes sapidus*): environmental and human health implications

**DOI:** 10.1007/s11356-025-36462-9

**Published:** 2025-05-02

**Authors:** Esperança Perelló, Samuel Pinya, Antoni Box, Antoni Sureda, Montserrat Compa

**Affiliations:** 1 Interdisciplinary Ecology Group, University of the Balearic Islands, 07122 Palma, Spain; 2 Health Research Institute of Balearic Islands (IdISBa), 07120 Palma, Spain; 3 Department of Agricultura, Ramaderia, Pesca, Caça i Cooperació Municipal, Consell Insular d’Eivissa, Eivissa, Ibiza, Balearic Islands 07800 Spain; 4 Research Group in Community Nutrition and Oxidative Stress (NUCOX), University of the Balearic Islands, 07122 Palma, Spain; 5 CIBEROBN (Physiopathology of Obesity and Nutrition), Instituto de Salud Carlos III, 28029 Madrid, Spain

**Keywords:** Toxicology, Bioindicators, Invasive species, Balearic Islands, Heavy metals, Human health

## Abstract

Heavy metals are increasingly becoming a significant environmental concern, especially regarding food safety and security. This is especially concerning as the consumption of harvested marine species can pose potential health concerns. The arrival of the blue crab, *Callinectes sapidus*, to the Balearic Islands has led to the need to evaluate its environmental implications and potential as a food source as part of management efforts. In the present study, the concentrations of cadmium (Cd), lead (Pb), and mercury (Hg) in the muscle tissue of *C. sapidus* were evaluated at several locations in the Balearic Islands in the western Mediterranean Sea. A total of eighty-three blue crabs were examined (45 males and 38 females) and the concentrations of the heavy metals were evaluated. Regarding metal concentrations, overall, Hg was the most present followed by Cd and then Pb. Concentrations were also site dependent, with Es Grau having the highest Hg concentrations while in s’Albufereta the highest Cd concentrations were observed. Moreover, differences in metal accumulation were also observed between sexes, with juveniles and females exhibiting higher Cd concentrations than males. Furthermore, regarding potential human health concerns for consumption, the margin of exposure (MOE) for Pb were all above 1 and the estimated weekly ingestion (EWI) for Cd and Hg, were all below the established guidelines for food safety. These results highlight that the consumption of *C. sapidus* from the Balearic Islands does not currently pose a health risk the recreational fisheries sector. Considering these findings, the muscle tissue of *C. sapidus* is a valuable bioindicator for monitoring heavy metal levels, and continued site-specific monitoring is recommended for the coastal ecosystems of the Balearic Islands.

## Introduction

Biological invasions threaten the structure and function of aquatic ecosystems on a global scale and continue to contribute to significant biodiversity loss and habitat degradation worldwide (Molnar et al. 2008). Addressing the effects of these invasions to develop management plans is particularly challenging in aquatic environments, as the pace at which invaders establish themselves often far exceeds the resources available for their control (Mooney and Cleland 2001; Zenetos 2017). Currently, in the Mediterranean Sea, invasive species have become a major concern, with the blue crab invasion emerging as a particularly problematic example.

The blue crab *Callinectes sapidus*, Rathbun 1896, is a decapod crustacean of the Portunidae family native to the western Atlantic, from Nova Scotia to northern Argentina (Wenner and Williams 1985). Its presence was first detected in the eastern Mediterranean in 1935, attributed to commercial interests (Giordani-Soika 1951; Galil et al. 2002). Since then, it has significantly expanded throughout the basin and has become one of the 100 worst invasive species (Zenetos et al. 2005). In Spain, it was first detected in the Tancada Lagoon, in the Ebro Delta (Catalonia) in 2012 (Castejón and Guerao 2013). Subsequently, the presence of *C. sapidus* spread rapidly along the Iberian Atlantic coast, reaching the southern coast of Portugal (Vasconcelos et al. 2019). In the Balearic Islands, the first report was recorded in the Albufera Natural Park (Mallorca) in the summer of 2017 (Garcia et al. 2018). That same summer, it was reported in the Albufera des Grau Natural Park and Cala Galdana stream systems (Menorca) (Garcia et al. 2018). The following summer, it was detected in the Ses Salines Natural Park of Ibiza and Formentera (Box et al. 2020), on both islands. Since its detection, the number of observations has been increasing, and since 2017, blue crab capture efforts have been underway at various sites across the Balearic Islands.

In its native range, *C. sapidus* holds significant commercial and recreational fishing value (Sharov et al. 2003), and as a result, its biology and ecology are well-documented. The diet composition of blue crabs generally includes mollusks (30–40%; mussels, clams, oysters), crustaceans (15–20%; decapods, amphipods), fish (15–20%), polychaetes (< 5%), and a variable percentage of algae, sediments, and detritus (Belgrad and Griffen 2016). In the Mediterranean Sea, *C. sapidus* has been identified as a carnivorous predator that shares trophic resources with benthic fish species in Croatia (Mancinelli et al. 2016). Its aggressive behaviour and opportunistic feeding habits lead to competition with native species and potential local extinctions (Marchessaux et al. 2023a).

The presence of *C. sapidus* in Mediterranean lagoons has raised concerns about its potential impacts on native biodiversity and artisanal fisheries (Marchessaux et al. 2023b), in addition to causing declines in commercial species and local biodiversity (Clavero et al. 2022). Although the economic consequences of its presence in the Mediterranean have not yet been extensively evaluated, is shown to prey on a wide variety of economically important species, including mollusks, fish, and crustaceans in Greek coastal areas (Kampouris et al. 2019), indicating a potentially substantial impact on regional fisheries and aquaculture. However, the exploitation of Mediterranean populations of *C. sapidus* is already under consideration from two perspectives: on the one hand, as a marine resource of high economic interest in its native regions in the USA, and on the other hand, as a management tool for a widely established species that could cause changes in habitat structure and associated biodiversity (Marchessaux et al. 2024). In the Mediterranean basin, it is commercially harvested in Turkey (Ayas and Ozogul 2011), northern Greece (Kevrekidis and Antoniadou 2018), and in Spain, in the Ebro Delta (Catalonia) (López and Rodon 2018).

Marine contamination by heavy metals has become a significant environmental concern. Since the onset of industrialization, large quantities of various chemical products have been released into the environment, altering the natural levels of these compounds (Briffa et al. 2020). The presence of heavy metals is particularly significant in aquatic habitats due to their environmental persistence, toxicity to living organisms, and bioaccumulation potential (Ali et al. 2019). Natural sources of heavy metals in the environment include rock weathering, volcanic eruptions, and sediment resuspension, whereas the primary anthropogenic sources encompass industrial emissions, mining, smelting, pesticide and phosphate fertilizer applications, biosolid usage (such as livestock manure, compost, or sewage sludge), landfill leachates, municipal and industrial wastewater, urban runoff, automobile emissions, and roadworks (Dixit et al. 2015; Briffa et al. 2020; Saravanan et al. 2024). Cadmium typically occurs in nature at low concentrations and is primarily generated as a by-product of zinc (Zn), copper (Cu), and Pb mining activities (Eisler 2000). Elevated concentrations of these elements have been detected in agricultural soils linked to the use of sewage sludge as fertilizer. Pb contamination in the environment has also been associated with the use of leaded gasoline, shotgun pellets, and fishing weights (Briffa et al. 2020). Pb is a non-essential, toxic metal whose biogeochemical cycle has been affected by human activities to a great degree (Komárek et al. 2008). Nowadays, atmospheric aerosols are the dominant source of Pb in the coastal marine environment and the open ocean (Chen et al. 2016). Hg primarily enters marine ecosystems through riverine inputs, which are a major source of Hg. Global Hg discharge from rivers into coastal oceans are estimated at 27 ± 13 Mmol year⁻^1^ (5500 ± 2700 Mg year⁻^1^), with 28% reaching the open ocean and the remainder depositing in sediments. Globally, riverine Hg inputs to the open ocean account for 30% of atmospheric contributions (Amos et al. 2014). Methylmercury (MeHg), a neurotoxin formed from inorganic Hg, bioaccumulates in aquatic food chains and adversely impacts human health globally through fish consumption (Mahaffey et al. 2011; Karagas et al. 2012). Health concerns regarding heavy metal pollution are well-documented. Numerous studies describe the adverse effects of consuming food with excessive levels of these and other toxic elements. Examples include irritation and renal failure (Tchounwou et al. 2012), neuronal damage (Ansari et al. 2003; Sanders et al. 2009), and carcinogenic effects (Clancy et al. 2012; Cohen et al. 2013).

Currently, the blue crab is not listed as an invasive species under Spanish Royal Decree 630/2013, regulating the Spanish Catalogue of Invasive Exotic Species. In Spain, the Ministry of Agriculture, Food, and Environment included it in the list of accepted commercial designations for fishing species (BOE-A-2016–3357). In the Balearic Islands, the *Consell Insular of Mallorca* authorized recreational blue crab fishing in eleven areas of Mallorca via a resolution (BOIB No. 205, December 8, 2020), whereas fishing is not currently permitted on the other islands.

Considering this, although *C. sapidus* has been extensively studied in terms of its biology, trophic interactions, and fisheries potential (Sharov et al. 2003; Marchessaux et al. 2024), there is a gap in information regarding its role as a potential bioindicator species for marine pollution, especially for the Mediterranean region. Furthermore, as it is from a higher trophic level and previous reports have identified it as a species that can bioaccumulate toxins in its system over time, the species could serve as a useful model for assessing heavy metal pollution in coastal ecosystems. However, to date, considering the potential for food safety as they are being harvested for human consumption, the concentrations of Cd, Pb, and Hg in *C. sapidus* from the Balearic Islands have not yet been evaluated.

To address these challenges, the study aimed to evaluate the potential of *C. sapidus* for assessing the concentrations of three heavy metals (cadmium, lead, and mercury) along the coastal areas of the Balearic Islands. The results of this research help identify the locations of *C. sapidus* for effective monitoring of marine environmental health, with implications for assessing risks related to human consumption.

## Materials and methods

### Study area

**Fig. 1:**
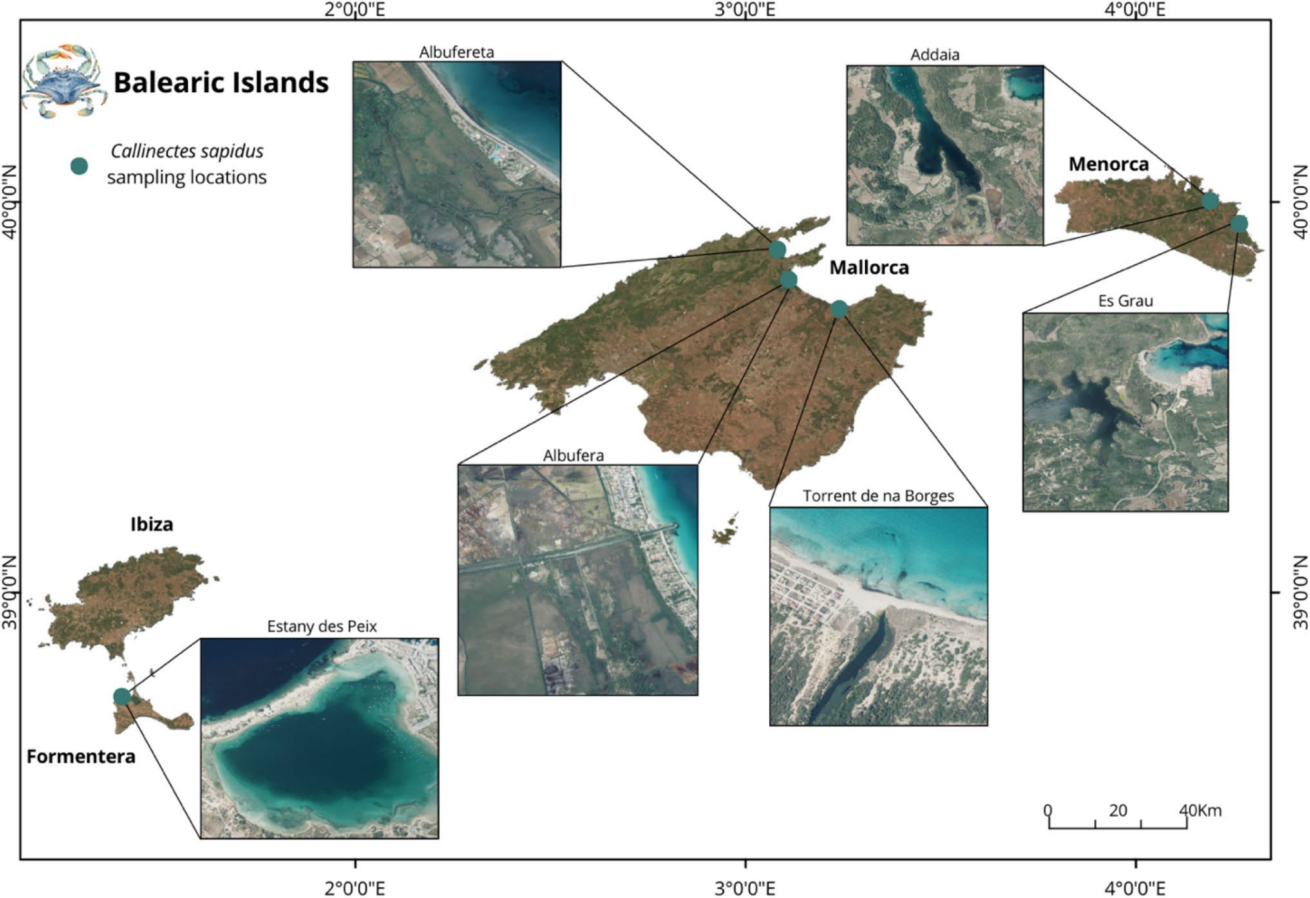
Map of the Balearic Islands showing *C. sapidus* sampling locations across Mallorca, Menorca, Ibiza, and Formentera. Satellite imagery insets highlight specific sampling sites along coastal areas and bays

The Balearic Islands are a current focal point for invasive species, especially in coastal ecosystems. This study surveyed three locations in Mallorca, two locations in Menorca, and one location on the island of Formentera to determine the accumulation of heavy metals in the muscle tissue of *C. sapidus* (Fig. 1). The first site was in the Albufereta of Pollença (39°51′46.98″N, 3°05′18.96″E), which is one of the typical lagoons in the western Mediterranean, located in the Bay of Pollença, in the northeast of Mallorca. It is characterized by a brackish marsh with ponds and canals separated from the sea by a bioclastic beach-dune system, which has an outlet that connects the lagoon with the sea, forming a micro-delta. The tides here are negligible, with most of the morphology and dynamic processes driven by wave activity and barometric pressure fluctuations (Pacheco et al. 1996). The second site in Mallorca is S’Albufera, and it is the largest wetland in the Balearic Islands, located in the northeast of Mallorca (39°47′20.47″N, 3°05′49.13″E). The hydrology of the wetland depends on the discharge of the northern mountain range torrents (Alcúdia basin, 620 km^2^), together with groundwater and seawater; there are no permanent watercourses on the island due to low rainfall and karst geology. Water contributions are irregular, as the surface rivers are of a stationary type (Alambiaga et al. 2024). The third location is the Torrent de Na Borges (39°43′28.16″N 3°14′06.29″E), located on Mallorca’s northern coast with an outlet in the Bay of Alcúdia, it is the island’s longest stream, spanning about 40 km and exhibiting a permanent water regime, fed by the aquifer and a 338 km^2^ hydrographic basin, the largest on Mallorca. The significant influx of freshwater forms a pond at its mouth with a natural outlet to the sea, resulting in periodic colonization by typical estuarine marine species. The estuarine crustacean fauna inhabiting this area is typical of these habitats (Garcia and Pinya 2022).

In Menorca, species were collected from two sites, the Albufera des Grau and within the Bay of Addaia. The Albufera des Grau (39°56′55.9″N, 4°15′04.2″E) is a brackish coastal lagoon located on Menorca’s northeast coast, irregularly connected to the sea. Freshwater inflows are primarily torrential, fed by two streams that drain a 56 km^2^ area. These freshwater inputs are currently highly intermittent, leaving the lagoon subject to substantial seasonal and interannual hydrological variability. This variability, mainly driven by water levels and salinity, is critical to the ecosystem’s ecological status. Unusual fluctuations in these hydrological parameters can drive the system into critical conditions with significant ecological impacts, such as haline stratification and subsequent hypoxia in the basin due to massive seawater inflow, mid-term salinization, which can lead to hypersalinity, and coastal desiccation, driven by falling water levels, which can cause large-scale die-off of macrophyte meadows throughout the lagoon. To avoid these undesirable situations, water exchange between the lagoon and the sea is regulated by two sluices. The system connects to the sea via a narrow, 500-m-long channel, where a small sluice (approximately 2 m^2^) regulates the lagoon-sea connection when the sand barrier at the end of the channel is opened (Obrador et al. 2008). The characteristics of the Bay of Addaia (39°59′30.1″N, 4°12′24.7″E) are slightly different. It is a relatively small, shallow (less than 12 m deep), and sheltered bay located on Menorca’s northern coast, where the seabed is largely covered by seagrass meadows (Ribera et al. 1997). The Mongofre–Addaia Meadows and Salt Pans comprise a shallow coastal area on the island’s northern coast, formerly used as salt pans until 1990. The main pan faces eutrophication and isolation due to fragmentation from the surrounding pans. The area features diverse habitats, including temporary ponds, meadows, freshwater ponds, tamarisk vegetation, drainage channels, and salinized lagoons. While most of the area stays flooded year-round, some zones experience temporary flooding. Marine influence shapes water composition, with freshwater inputs from the Mongofre and Cala Addaia streams. There are point-source pollution spots due to nearby urban development. Currently, a dike separates the wetland from the sea (WWF España 2018). Finally, the sixth location was the Estany des Peix in Formentera (38°43′32.48″N, 1°24′43.34″E). This lagoon is within the scope of the Ses Salines Natural Park of Ibiza and Formentera. It also belongs to the Natura 2000 network as an SPA under the designation ES0000084 Ses Salines d’Eivissa i Formentera (Table 1).

**Table 1:** Summary of the sampling locations and a description of the physical characteristics and protection status of the sampling locations

Island	Sampling site	Physical characteristics	Habitats	Protection status
Mallorca	Albufereta	Wetland	Grasslands and marshlands	Special Protection Area of Birds; Natural Reserve
	Albufera	Coastal lagoon	Grasslands and marshlands	Special Area of Conservation, RAMSAR wetland, Natural Park
	Torrent de Na Borges	Coastal lagoon	Wet meadows, dunes	Special Area of Conservation; Natural Park
Formentera	Estany des Peix	Coastal lagoon	Grasslands and marshlands	RAMSAR wetland, Natural Park
Menorca	Es Grau	Coastal lagoon	Grasslands and marshlands	Biosphere Reserve; Special Area of Conservation; Special Protection Area of Birds; Natural Park
	Addaia	Wetland	Grasslands and marshlands	Biosphere Reserve; Special Area of Conservation; Special Protection Area of Birds; Natural Park

### Sampling and sample preparation

A total of 83 muscle tissues of *C. sapidus* were collected and analyzed for heavy metal concentrations. The collection methods varied depending on the area from which specimens were obtained. Specimens from protected natural areas were captured as part of the routine work conducted by the management authorities responsible for these areas. These include S'Albufereta de Pollença (Mallorca), S’Albufera de Mallorca Natural Park (Mallorca), and Estany des Peix (Formentera), in addition to Addaia (Menorca) and Albufera des Grau (Menorca). Specifically, in S’Albufera de Mallorca Natural Park, captures of *C. sapidus* were incidental and part of the population control measures for the invasive species *Trachemys scripta* Schoepff, 1792. For this purpose, traps adapted for the invasive turtle were used, consisting of truncated cone shapes with an entry opening at one end, 86 cm in total length, and a mesh size of 1 cm. In the case of Torrent de Na Borges (Mallorca), the captures formed part of a study examining the species’ colonization process along the Balearic coasts. Samples were collected between summer 2019 and winter 2021 (in S’Albufereta and Torrent de Na Borges, summer 2019; in S’Albufera de Mallorca, Es Grau, and Estany des Peix, summer and autumn 2020; and in Addaia, winter 2020–2021).

For each specimen, sex (male/female), maturity status (adult/juvenile), and biometric data, such as carapace width (CW) and wet weight (WW), were recorded to assess potential correlations with heavy metal content. Muscle tissue samples were taken from one of the legs and frozen at − 20 °C for later analysis. Sampling muscle tissue from the legs of marine species is a standardized practice for assessing the risks associated with their consumption, as it provides relevant data regarding bioaccumulation of contaminants and human dietary exposure.

To prepare the samples, 0.4 g aliquots of fresh muscle tissue from each individual were dried over three days at 60 °C until a constant weight was obtained. The dried tissues were digested using 8 mL of 65% nitric acid (HNO₃) and 2 mL of hydrogen peroxide (H₂O₂). Samples were kept covered with opaque material to prevent light penetration during this process. After digestion, the samples were filtered with 0.45 µm filters to remove impurities and subsequently analyzed for Cd, Hg, and Pb content. To minimize contamination, all samples were handled with powder-free nitrile gloves, and stainless-steel instruments were used to avoid cross-contamination. Muscle tissue samples were dissected under clean conditions using acid-washed stainless-steel scalpels and stored in polyethylene containers previously rinsed with ultrapure water. Samples were immediately frozen at − 20 °C until further processing. Furthermore, prior to dissection, all laboratory equipment and containers were pre-cleaned with nitric acid (10%) and rinsed with deionized water to remove potential trace metal residues.

### Determination of metal concentrations

Quantitative determinations of heavy metals were conducted using inductively coupled plasma mass spectrometry (ICP-MS) (Agilent Technologies model 7700x, Santa Clara, CA, USA), with scandium, germanium, rhodium, and rhenium used as internal standards at 500 ppb. The sample introduction system for this instrument employed the high-temperature torch-integrated sample introduction system (hTISIS) along with a high-efficiency nebulizer (HEN, Meinhard Glass Products, Golden, CO, USA). Calibration standards at varying concentrations (0, 0.5, 1, 5, 10, 50, 100, and 500 ppb) were prepared using certified reference material (Multielement Std, CP33MS, SCP Science, Canada). Each injection was measured three times, and the calibration curve was established with an *r*^2^ ≥ 0.995. Blank samples with Millipore water and blank spikes were also included to ensure quality control. The detection limit was set at three times the standard deviation of the blank samples and was below 0.001 ppb for the elements analyzed. Chemical blanks from the experimental procedure were less than 2% of the sample signal and were used to correct the sample results. For quality control, DORM-4 fish protein from certified reference material (CRM) by the National Research Council of Canada (NRCC) was used.

### Health risk assessment

One management strategy for controlling the population of *C. sapidus* as previously stated, is its capture for commercial and recreational consumption. However, it is crucial to evaluate potential health risks, such as the ingestion of heavy metals that may accumulate in muscle tissue. This analysis was done considering the following indicators: the proportion of samples with heavy metal content above legal limits in Europe (Cd, Hg, and Pb); the Tolerable Weekly Intake (Cd and Hg); and the Margin of Exposure (Pb).

#### Legal content limits

The European legislation, specifically the Commission Regulation (EU) 2023/915 (available at https://eur-lex.europa.eu/legal-content/EN/TXT/HTML/?uri=CELEX:32023R0915, accessed October 2024), regulates the maximum quantity of a contaminant allowed for each food type, which in the case of crustaceans is 0.50 mg/kg for the three metals considered.

#### Tolerable weekly intake values (TWI)

The tolerable weekly intake values (TWI) recommended by the European Food Safety Authority (EFSA) are 2.5 μg/kg bw/week for Cd (European Food Safety Authority 2010), and 1.3 μg/kg bw/week for MeHg (European Food Safety Authority 2012). When the weekly intake of a product is higher than those limits, it poses a risk to human health if consumed.

The Estimated Weekly Intake (EWI, μg/kg body weight/week) depends on several factors, including metal concentration, food consumption, and body weight, and is calculated using the formula:

$$EWI=(Cm\times FIR)/ABW$$

where Cm represents the heavy metal concentration present in the sample (μg/kg wet weight), FIR (food intake rate) indicates the average weekly consumption of crab in the local area, which is 5.4 kg/year per capita = 103.85 g/week in the Balearic Islands (Ministry of Agriculture, Fisheries and Food 2024). It should be borne in mind that the data used corresponds to the global consumption of shellfish, molluscs, and crustaceans and that this category includes clams, cockles, mussels, squid, octopus, prawn/shrimp, and other shellfish/molluscs/crustaceans. Finally, ABW represents average body weight. We used López-Sobaler et al. (2016) as a reference for the Spanish population: 82.4 kg for men and 66.6 kg for women, with an average of 74.5 kg for adults. If the calculated EWI exceeds the TWI, a product can be considered unsafe.

#### Margin of exposure (MOE)

Carcinogenic substances (like Pb) safety limits can be expressed as Benchmark Dose Lower Limits (BMDL). If the MOE value is lower than 1, it represents a potential concern for human health (European Food Safety Authority 2005). The value used for Pb is 0.63 μg/kg/day (European Food Safety Authority 2010). MOE (dimensionless) was calculated using the formula:

$$MOE=(BMDL/C)\times (DFI/ABW)$$

where *C* represents the concentration of Pb present in the sample (μg/g), DFI is the daily intake, which is equivalent to 14.84 g/day in Spain as seen above and ABW is the average body weight of 74.5 kg per adult.

### Data analyses

Data analyses were performed to determine differences in concentrations of Cd, Pb, and Hg considering condition factors, sex, and location. Furthermore, the condition factor *K* was calculated using the following formula: *K* = 100 *W/*CW*^3^, where *W* is the weight of the crab (in grams) and *CW* is the carapace width.

In the present study, Pearson’s correlation was employed to examine the linear relationship between the condition factor and heavy metal concentrations in the samples. This method was selected to assess the strength and direction of association between these variables, assuming linearity and measurement on continuous scales. To determine significant differences between the heavy metals in each sex, pairwise comparisons were conducted using the Mann–Whitney *U*-test. Regarding heavy metals in muscle tissue according to locations, a Kruskal–Wallis test was applied for each location and then by each sex separately. Furthermore, if significance was determined, a Dunn’s test was performed to provide pairwise comparisons between the coefficients. Furthermore, outliers in the concentrations of Cd, Pb, and Hg were identified using the interquartile range (IQR) method, and those containing outliers in any of the metals were excluded from the final dataset to ensure the analysis was based on non-extreme values. For all data analyses, normality was determined based on the Shapiro–Wilk test, and the homogeneity of the variances was determined using Levene’s test. Non-parametric tests were performed as assumptions for parametric tests were not met. For all tests, significance was set at *p* < 0.05. R version 4.4.2 was used for the statistical analysis.

## Results

### Species biometrics

A total of 83 samples of crabs caught in six locations in the Balearic Islands were analyzed. The samples correspond to 12 females in S’Albufereta and Torrent de Na Borges, 12 males in Estany des Peix and Es Grau, 15 males and 9 females in S’Albufera, and 6 males and 5 females in Addaia (Table 2). The average carapace width of the females was found to be 13.1 ± 2.5 cm, weighing an average of 123.3 ± 57.9 g (wet weight), and the males were slightly larger with an average carapace width of 14.5 ± 1.7 cm, weighing an average of 229.7 ± 74.7 g (wet weight). According to the locality, the largest individuals were those caught in S’Albufera in Mallorca, followed with little difference by those from Es Grau in Menorca, while the smallest were those from S’Albufereta in Mallorca (Table 1). It should be noted that the distribution of sexes and life stages was not uniform across locations. For this reason, S’Albufereta samples were treated as a separate case study as only juvenile females were captured.

**Table 2:** Summary of the biometrics for the female and male *Callinectes sapidus* collected at each of the six sampling sites distributed throughout the Balearic Island

Sampling site	Island	Female			Male		
		*n*	Width (cm)	Weight (g)	*n*	Width (cm)	Weight (g)
Albufereta	Mallorca	12	10.2 ± 0.6	59.7 ± 16.7			
Albufera	Mallorca	9	15.7 ± 2.4	182.0 ± 55.2	15	14.8 ± 1.8	199.3 ± 61.3
Torrent de Na Borges	Mallorca	12	14.3 ± 1.3	144.2 ± 31.1			
Estany des Peix	Formentera				12	14.2 ± 1.3	247.5 ± 62.1
Es Grau	Menorca				12	15.0 ± 2.0	271.2 ± 92.1
Addaia	Menorca	5	14.0 ± 0.4	120.0 ± 15.8	6	13.3 ± 1.0	187.3 ± 40.9
Total		32	13.1 ± 2.5	123.3 ± 57.9	45	14.5 ± 1.7	229.7 ± 74.7

The biometrics include carapace width (cm) and wet weight (g) for the mean and standard deviation

### Heavy metal concentrations

Heavy metals were observed in the muscle tissue in all 83 individuals for *C. sapidus* analyzed from the Balearic Islands overall, an average ± standard deviation of 0.06 ± 0.09 μg/g of Cd, 0.06 ± 0.04 μg/g of Pb and an average of 0.21 ± 0.14 μg/g of Hg was observed in all samples.

**Fig. 2:**
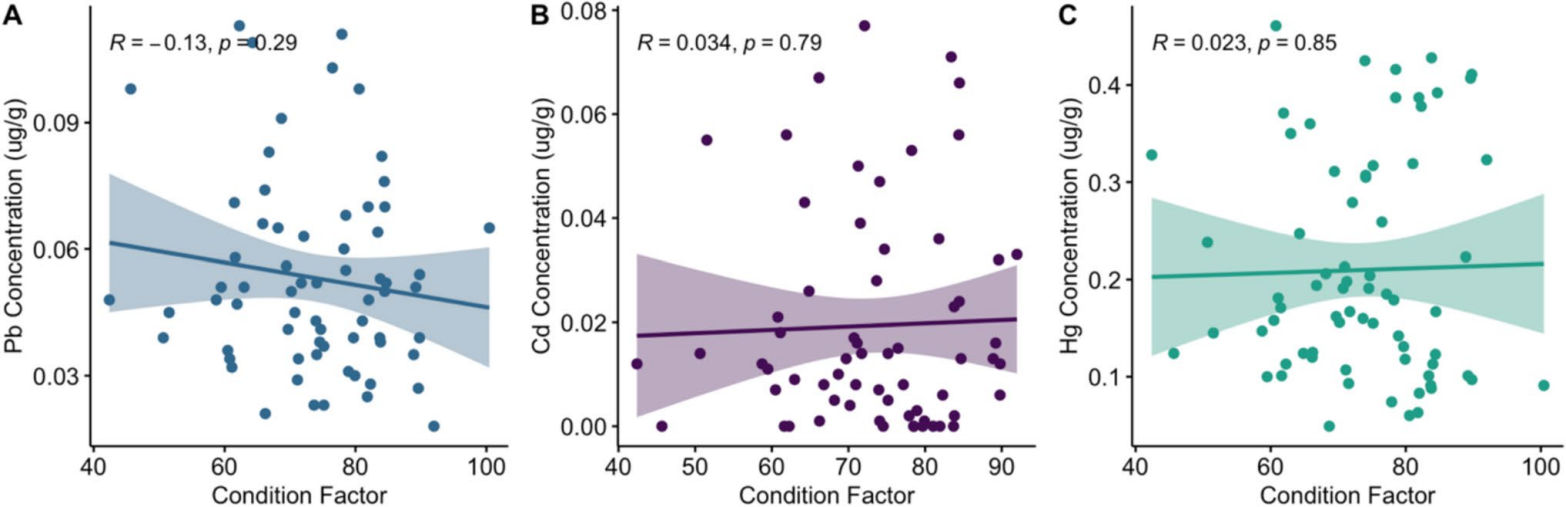
Relationships between condition factor and metal concentrations (µg/g) for **A** lead (Pb); **B** cadmium (Cd); and **C** mercury (Hg). Correlation coefficients (*R*) and *p*-values are provided for each plot. Shaded areas represent the 95% confidence intervals

The analysis of the relationship between the condition factor and the concentrations of Pb, Cd, and Hg revealed no significant correlations (Fig. 2). For Pb, the correlation was weakly negative, indicating no significant association, and Cd and Hg showed very weak positive correlations. These results suggest that the condition factor does not significantly influence the concentrations of these metals in the sampled organisms, with all relationships being statistically nonsignificant.

Analysis of heavy metal concentrations in *C. sapidus* across the Balearic Islands revealed distinct patterns between locations and sexes (Table 3). From a total of 83 specimens (38 females and 45 males) collected across six sampling sites, females showed higher average Cd concentrations (0.08 ± 0.11 μg/g) compared to males (0.03 ± 0.05 μg/g), with the highest levels recorded in females from Albufereta (0.18 ± 0.12 μg/g). For males, significant differences were observed between Es Gray and Estany des Peix (Table 3; KW, *p* < 0.001). Hg concentrations were comparable between females (0.22 ± 0.09 μg/g) and males (0.21 ± 0.18 μg/g), with peak levels significantly higher in males from Es Grau (0.37 ± 0.05 μg/g) compared to Addaia, Albufera and Estany des Peix (Table 3; KW, *p* < 0.01). females from Albufera (0.31 ± 0.12 μg/g). Pb concentrations remained relatively consistent between sexes (females: 0.06 ± 0.03 μg/g; males: 0.07 ± 0.04 μg/g). It is important to highlight that in some locations, specimens of only one sex were sampled, for example in Albufereta and Torrent de Na Borges there were only females, while in Estany des Peix and Es Grau, only males were captured. The most comprehensive sampling was achieved at Albufera and Addaia, where both sexes were represented, allowing for direct comparisons of metal accumulation patterns between males and females at these locations.

**Table 3:** Metal concentration in muscle tissue of specimens of *Callinectes sapidus* from the present study

Sampling Site	Island	Female				Male			
		*n*	Cd (μg/g)	Hg (μg/g)	Pb (μg/g)	*n*	Cd (μg/g)	Hg (μg/g)	Pb (μg/g)
Albufereta	Mallorca	12	**0.18 ± 0.12**^**&%**^	**0.17 ± 0.03**^**$**^	**0.09 ± 0.03**^**&**^				
Albufera	Mallorca	9	0.09 ± 0.14	0.31 ± 0.12	0.06 ± 0.02	15	0.02 ± 0.02	0.12 ± 0.03	0.08 ± 0.06
Torrent de Na Borges	Mallorca	12	0.02 ± 0.02	**0.19 ± 0.06**^**$**^	0.04 ± 0.01				
Estany des Peix	Formentera					12	**0.09 ± 0.08**^**@%$**^	0.19 ± 0.29	0.06 ± 0.02
Es Grau	Menorca					12	0.01 ± 0.01	**0.37 ± 0.05**^**#%$**^	0.05 ± 0.02
Addaia	Menorca	5	0.02 ± 0.01	0.23 ± 0.06	0.06 ± 0.03	6	0.02 ± 0.02	0.17 ± 0.05	0.06 ± 0.05
Total		**32**	**0.08 ± 0.11**	**0.22 ± 0.09**	**0.06 ± 0.03**	**45**	**0.03 ± 0.05**	**0.21 ± 0.18**	**0.07 ± 0.04**

Values are reported as μg/g of wet weight and are expressed as mean ± standard deviation. Significant differences are represented as ^&^with respect to Torrent de na Borges; ^#^with respect to Estany des Peix; ^%^with respect to Addaia; ^$^with respect to Albufera; and ^@^with respect to Es Grau, and significance was set *p* < 0.05

**Fig. 3:**
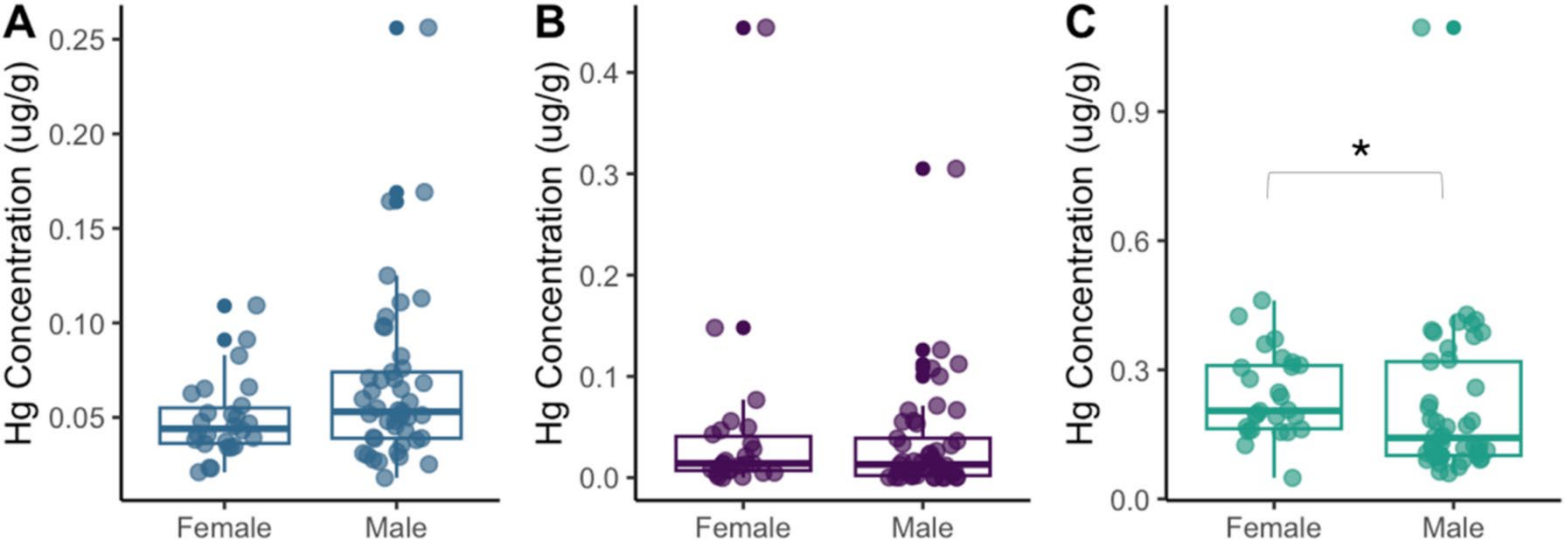
Concentration of the heavy metals determined by the sex (female/male) of *Callinectes sapidus* in muscle tissue for **A** lead (Pb); **B** cadmium (Cd); and **C** mercury (Hg) expressed in ug/g. Concentration is expressed as the mean and error bars are the standard errors. *indicates significance at *p* < 0.05

Excluding S’Albufereta, the statistical analysis of heavy metal concentrations between male and female *C. sapidus* was element specific (Fig. 3). Pb concentrations showed no significant differences between males (0.07 ± 0.04 μg/g) and females (0.05 ± 0.01 μg/g) (MW, *p* > 0.05). Cd concentrations were slightly higher in females (0.04 ± 0.02 μg/g) compared to males (0.03 ± 0.05 μg/g) (MW, *p* > 0.05). Finally, Hg levels were significantly different between sexes (MW, *p* < 0.05), with females showing higher concentrations (0.24 ± 0.10 μg/g) than males (0.21 ± 0.18 μg/g).

**Fig. 4:**
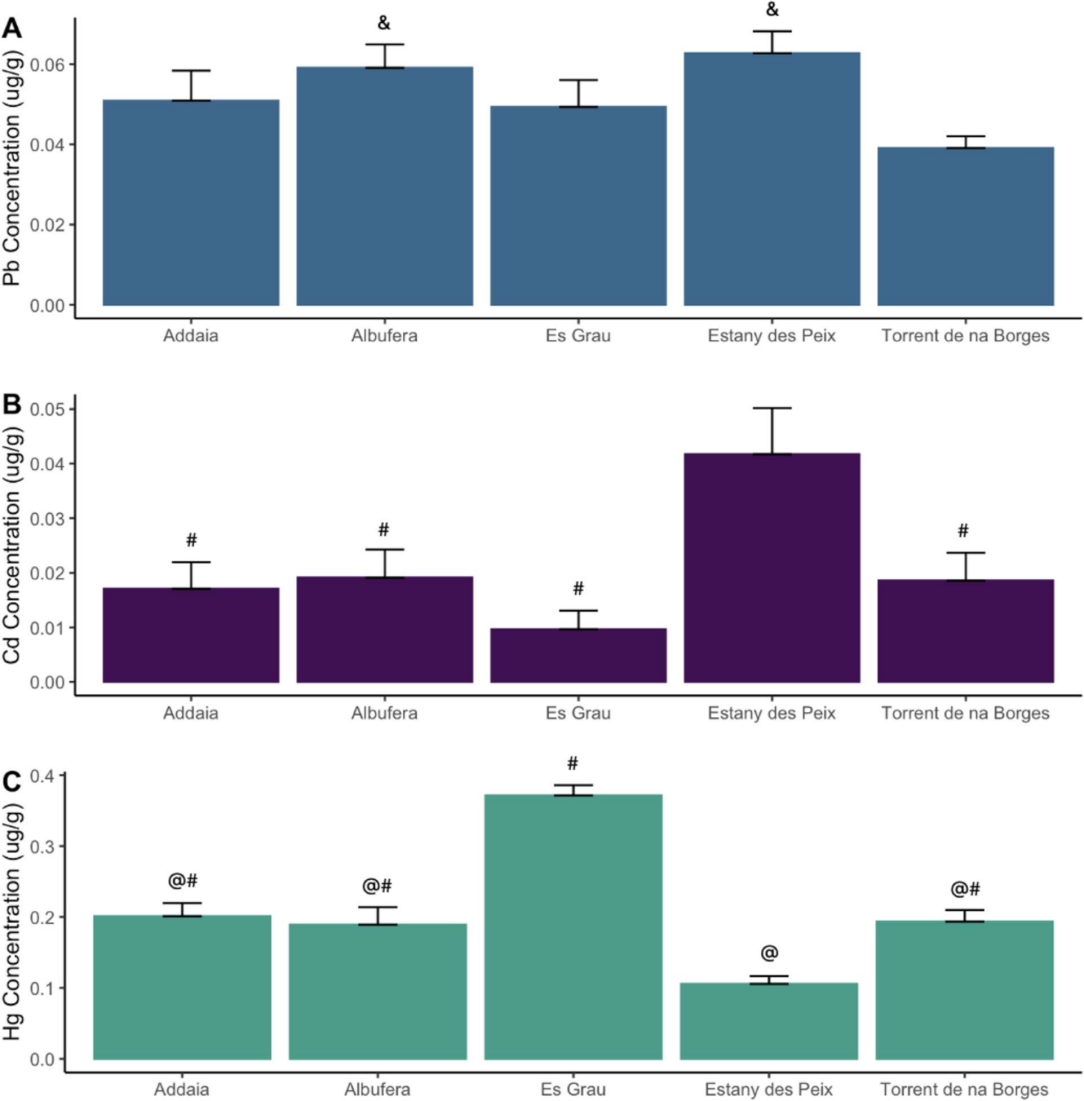
Concentration of the heavy metals determined by the six sampling sites of *Callinectes sapidus* in muscle tissue for **A** lead (Pb); **B** cadmium (Cd); and **C** mercury (Hg) expressed in ug/g. Concentration is expressed as the mean and error bars are the standard errors. Significant differences are represented as ^&^with respect to Torrent de na Borges; ^#^with respect to Estany des Peix; ^%^with respect to Addaia; and.^@^with respect to Es Grau, and significance was set at *p* < 0.05

In terms of concentrations of heavy metals by site, significant differences were observed between Pb between Albufera (KW, *p* < 0.05) and Estany des Peix (KW, *p* < 0.001) compared to Torrent de na Borges. Furthermore, significant differences were observed in Cd between all locations and Estany des Peix in Formentera (Fig. 4B, KW, *p* < 0.05). Regarding Hg, on the other hand, significant differences were observed between all Es Grau and all other sites, where the highest concentration of Hg in muscle tissue was observed (Fig. 4C, KW, *p* < 0.05). Significant differences between Estany des Peix and all other sites were observed, with the lowest concentrations reported in samples collected at this location (Fig. 4C, KW, *p* < 0.05).

### Case study Albufereta

Regarding the individuals sampled in Albufereta, the highest concentrations were observed in S’Albufereta (0.18 ± 0.12 µg/g) and for Pb (0.09 ± 0.03 µg/g). While only 0.17 ± 0.03 µg/g of Hg was reported in the tissue. Previous studies have observed Hg primarily accumulates in muscle tissue while Pb and Cd observed an opposite pattern, where higher concentrations can be observed in whole-body tissue compared to muscle tissue (Adams and Engel 2014). Furthermore, accumulation dynamics are metal dependent, in Adams and Engel (2014), the study reported Cd and Pb levels decreased when *C. sapidus* moved to cleaner environments while the Hg levels remained the same.

### Health risk assessment

#### Legal content limits

Although heavy metals were observed in all the studied individuals, no samples exceeded the maximum permissible levels for crustaceans, set at 0.50 mg/kg by the European Commission (2023). The highest concentration recorded was for Hg in Es Grau, at 0.37 μg/g. However, it is noted that when considering the values by sex, the female specimens from S’Albufera also show concentrations of the same order as those from Es Grau (where only male samples were observed), reaching values of 0.3 μg/g.

#### Tolerable weekly intake (TWI) values

TWI values by sampling site are presented in Table 4. To obtain a more accurate estimate that accounts for individual sample variability, TWI was calculated for each sample, with the mean then calculated per site. For this study, cadmium (Cd) and mercury (Hg) did not exceed safety thresholds, which are set at 2.5 μg/kg body weight/week for Cd and 1.3 μg/kg body weight/week for methylmercury (MeHg).

**Table 4:** Estimated weekly ingestion (TEWI, in μg/kg body weight/week) of Cd and Hg detected in all the samples and Margin of exposure (MOE) for Pb

Sampling Site	Island	*n*	EWI (Cd)	EWI (Hg)	MOE (Pb)
Albufereta	Mallorca	12	0.25 ± 0.16	0.24 ± 0.05	1.53 ± 0.58
Albufera	Mallorca	24	0.06 ± 0.13	0.26 ± 0.17	2.34 ± 1.2
Torrent de Na Borges	Mallorca	12	0.03 ± 0.02	0.27 ± 0.08	3.47 ± 1.14
Estany des Peix	Formentera	12	0.12 ± 0.11	0.26 ± 0.40	2.24 ± 0.97
Es Grau	Menorca	12	0.01 ± 0.02	0.52 ± 0.07	3.13 ± 1.59
Addaia	Menorca	11	0.02 ± 0.02	0.28 ± 0.08	2.65 ± 1.11

Results are presented as means ± SD

EWI values for Cd were consistently low across all sites, with the highest level observed at Albufereta (Mallorca) (0.25 ± 0.16 μg/g). While variability across sites is notable, most Cd levels remain well below the recommended TWI threshold of 2.5 μg/kg body weight/week. In contrast, Hg levels were generally higher than those of Cd, with Es Grau (Menorca) showing the highest mean concentration at 0.52 ± 0.07 μg/g. This elevated concentration may indicate a higher exposure risk in this area, although EWI values for Es Grau remain within established safety limits for Hg.

#### Margin of exposure (MOE)

Regarding the MOE values for lead (Pb) (Table 4), MOE values were calculated for each sample to account for within-site variability, following the same approach used for EWI. An MOE of 1 or higher is generally considered acceptable, with higher values indicating a lower potential health risk. The highest MOE values for Pb were observed in Torrent de Na Borges (Mallorca) and Es Grau (Menorca), with values of 3.47 ± 1.14 and 3.13 ± 1.59, respectively, suggesting a low health risk at these sites. In contrast, Albufereta showed the lowest MOE (1.53 ± 0.58), indicating a relatively higher exposure to Pb in this area, which may warrant closer monitoring as it approaches the minimum safety threshold.

Results are presented as means ± SD.

## Discussion

This study examined the accumulation of lead (Pb), cadmium (Cd), and mercury (Hg) in the muscle tissue of the invasive crab *C. sapidus* in the Balearic Islands, located in the western Mediterranean Sea. While all specimens tested contained detectable levels of these metals, concentrations were within the safety thresholds established by the European Union (EU) for human consumption.

For the first time, metal concentrations are reported in this study for the Balearic Islands, although there are a few studies conducted in different locations within the Mediterranean basin (Table 5). In general, Cd and Pb concentrations in the Balearic samples were lower and site and sex specific. Regarding Cd, values that exceeded some of those recorded in the previous studies were females in S’Albufereta (0.18 μg/g), higher than the 0.03 μg g⁻^1^ obtained in Loudias (Greece) and the 0.16 μg g⁻^1^ obtained in Aquatina (Italy) by De Giorgi et al. (2024), as well as those obtained in Sicily by Di Salvo et al. (2024) (0.13 μg g⁻^1^) or by Agilkaya et al. (2022), which were below the detection limits. Regarding Pb, the concentrations were lower than those recorded in any of the other locations in the Mediterranean basin. These differences may be attributed to the lower intensity of pollution sources in the Balearic Islands, as there are no large-scale industries, and the existing ones are predominantly located far from the coastline. Additionally, variations in local habitats and the specific exploitation and consumption practices characteristic of this region could also play a role. In contrast, Hg levels, particularly at Es Grau (0.37 μg/g), were significantly higher than those reported elsewhere. This difference highlights the unique environmental conditions of Es Grau and their potential to enhance MeHg production. The high levels observed align with the findings by Al-Sulaiti et al. (2022), who identified similar dynamics in areas with rich organic sediments. Further studies, such as those by Liu et al. (2019), emphasize the role of anaerobic conditions in mercury methylation, suggesting that Es Grau’s ecosystem might favor this transformation more than other studied sites. Overall, *C. sapidus* presents itself as a unique bioindicator to compare for metal concentrations across the Mediterranean Sea.

**Table 5:** Comparison of the mean metal concentrations in *Callinectes sapidus* with results taken from the other studies from the Mediterranean Sea

Reference	Location	Cd	Hg	Pb	Content
Younis et al. (2024)	Temsah Lake, Egypt, Winter	0.58 ± 0.12	0.011 ± 0.005	5.7 ± 0.39	Evaluate seasonal variation in the concentration of metals in several species of fish and crustaceans, including the blue crab
	Temsah Lake, Egypt, Summer	0.69 ± 0.19	0.004 ± 0.001	7.8 ± 0.41	
Di Salvo et al. (2024)	Maragani River, Sicily	0.13 ± 0.01	0.04 ± 0.01	0.19 ± 0.03	Analysis of 23 minerals in blue crabs captured in two different locations
	Adriatic coast area, Italy	0.11 ± 0.02	0.04 ± 0.01	0.21 ± 0.02	
De Giorgi et al. (2024)	Lesina, Italy	0.24 ± 0.04	-	0.17 ± 0.03	Concentration of 13 trace metals (B, Ba, Cd, Cr, Cu, Fe, Li, Mn, Ni, Pb, Sr, V and Zn) in blue crabs from five coastal locations in Spain, Italy and Greece
	Acquatina, Italy	0.16 ± 0.03	-	0.53 ± 0.06	
	Gandia, Spain	0.26 ± 0.09	-	0.64 ± 0.05	
	Pogonitsa, Greece	0.47 ± 0.06	-	0.22 ± 0.02	
	Loudias, Greece	0.03 ± 0.01	-	0.51 ± 0.04	
Agilkaya et al. (2022)	Göksu Delta, Turkey	< 0.0004		< 0.0003	Seasonal variations and the influence of sex on the levels of Fe, Cu, Zn, As, Cd and Pb in muscle tissue

Values are reported as micrograms per gram of wet weight and are expressed as mean ± SD

In terms of site-specific concentrations, notable geographical variations in metal concentrations were observed across the sampling sites. Cd levels peaked at S’Albufereta (0.18 μg/g), suggesting the influence of agricultural runoff and drainage, since the area is bordered by agricultural fields. Similarly, Pb concentrations were highest in S’Albufereta (0.06 μg/g) and lowest in the Torrent de Na Borges, potentially reflecting differences in land use and pollution sources. In the S’Albufereta area, intensively cultivated land is concentrated, while in the Torrent de Na Borges area, the land is of extensive use, with the predominant use being land dedicated to rainfed crops.

From an ecological perspective, wetlands such as Es Grau and S’Albufereta, exhibit characteristics that make them critical points for the accumulation of heavy metals (Liu et al. 2019). Sediments rich in organic matter and anaerobic conditions favor the conversion of inorganic mercury into MeHg by microbiological action, a process that contributes to bioamplification through the food web (Okeke et al. 2024). In the case of Es Grau, *C. sapidus* could be consuming prey located at higher trophic levels, which increases exposure to MeHg. These dynamics could also apply to S’Albufereta, where the levels of Cd and Pb were significantly higher than in other locations, suggesting the presence of mechanisms similar to those observed for Hg in Es Grau. The observed disparities in metal accumulation could stem from hydrodynamic variability, such as the higher flushing rates at Torrent de Na Borges compared to the stagnant conditions in S’Albufereta and Es Grau. Additionally, sediment type plays a crucial role; sandy sediments in Torrent de Na Borges may facilitate metal mobility, while organic-rich sediments in Es Grau and S’Albufereta enhance metal binding and methylation (Taylor and Calabrese 2018). These environmental differences underscore the need for site-specific pollution management strategies. In addition, historical factors, such as natural deposits of metals or ancient pollution sources, could have contributed to current concentrations, gradually releasing pollutants from the sediments. These patterns could be influenced by local environmental factors, such as sediment characteristics, water circulation, and potential point sources of contamination. For example, organic-rich sediments can bind metals more strongly while sandy sediments may facilitate the uptake of metals due to their low organic matter content (Taylor and Calabrese 2018). Furthermore, areas with well-flushed areas such as in the Torrent de na Borges, may show lower concentrations compared to the lagoons and wetlands where water circulation may be lower, increasing metal accumulation (Younis et al. 2024). Another potential source is pointing source pollutants, such as runoff from agriculture or wastewater discharge (Gutiérrez-Peña et al. 2018). Furthermore, an initial analysis to determine the potential for land cover was inconclusive and further would be recommended, especially in areas such as S’Albufereta where elevated concentrations were observed.

Sex- and age-based differences in metal accumulation were apparent in the sampled crabs. Females exhibited higher Cd levels, potentially due to reproductive or metabolic factors related to the life cycle (Adams and Engel 2014). Juveniles showed elevated concentrations of both Cd and Pb, which could be linked to their more active metabolism, distinct dietary habits, or underdeveloped detoxification systems. Similar trends have been reported in other aquatic species, such as *Squalius cephalus* Linnaeus, 1758, where juveniles exhibited higher heavy metal concentrations linked to diet and metabolic rate (Nyeste et al. 2019). Understanding these biological influences is essential for assessing both the ecological impacts and the sustainability of *C. sapidus* populations and their use as a fishery resource, requiring future research to clarify the underlying factors.

Although none of the analyzed samples exceeded EU safety limits, our findings do raise some concerns. The Hg levels at Es Grau approached the TWI threshold, posing potential risks with prolonged or frequent consumption. In this study, Hg in *C. sapidus* muscle tissue was analyzed, although mercury toxicity is primarily linked to its organic form, methylmercury (MeHg). Previous studies have suggested that MeHg represents a significant proportion of total Hg in marine organisms, typically ranging from 50 to 90%, depending on the study species and trophic level. In crustaceans, MeHg concentrations have been reported to vary between < 0.002 and 0.0221 µg/g, and although it is lower than in fish, it is still important to monitor due to biomagnification processes (Yu et al. 2020). Given that the TWI is established for MeHg, and not total Hg, our health risk assessment may result in higher risk estimates relative to actual MeHg. Future studies should incorporate speciation analyses to differentiate MeHg from inorganic Hg and refine risk estimates. Furthermore, in terms of MOE, the MOE for Pb at S’Albufereta (1.53 ± 0.58 MOE) was close to the risk threshold, emphasizing the need for continuous monitoring. In addition to the metals reported in this study, it would be important to include other elements such as Zn and Cr as they are also relevant for human health concerns considering their toxicity. A recent study by De Giorgi et al. 2024 highlighted their presence in *C. sapidus* from Spain, Italy and Greece, although their concentrations were significantly lower than in bivalves and lower than reference levels for human consumption. This suggests that despite the detection of several metals, the ecotoxicological risk is low although in large part it site-dependent and future monitoring is needed. In addition, the TWI and MOE, many countries have their own safety standards, there are a few international safety standards, such as those set by the Codex Alimentarius Commission (CAC) and World Health Organization (WHO), which establish maximum levels (MLs) for heavy metals like Hg, Cd, and Pb in food and water to protect public health and facilitate international trade. Although for *C. sapidus*, guidelines are not specifically set, the CAC does specify limits of 0.5–1.0 mg/kg for fish depending on the species while the WHO has set 6 µg/L for Hg, 3 µg/L for Cd, and 10 µg/L for Pb.

Currently in the Mediterranean Sea, the General Fisheries Commission for the Mediterranean (GFCM) (Food and Agriculture Organization of the United Nations) has implemented a regional research program which aims to establish a basin-wide framework for stock assessment of *C. sapidu*s in addition to the blue swimming crab *Portunus segnis* (Forskål, 1775), and its incorporation within fisheries due to its potential for both economical and nutritional value (Arena et al. 2024). To date, the ongoing invasion of the Atlantic blue crab in the Mediterranean Sea presents both challenges and opportunities, requiring a balanced approach to management that combines mitigation efforts with economic exploitation, enhancing a need for a strategy that aims to control the invasive populations while creating value for local communities (Mancinelli et al. 2017a). While the commercialization of invasive species for human consumption is an established approach in fisheries management globally (de Carvalho-Souza et al. 2024), its application to European waters presents unique challenges. The potential economic opportunities from managing invasive species through commercial harvest in European waters remain largely unexplored, primarily due to a limited understanding of their ecological impacts in these ecosystems. In countries such as Tunisia, one example of a management effort included using blue crab meal as a substitute in the manufacturing of aquaculture feed for fish farms and crab exports increased drastically from 38.4 tons in 2015 to 755.5 tons in 2017 (Mili 2021). Despite this, a significant challenge that remains in fisheries management is the economic viability of blue crab exploitation, as the species'low meat yield in combination with high processing costs can make it difficult to commercialize at a large scale (Arena et al. 2024). In Marchessaux et al. (2024), a pilot survey in France reported they were keen to include *C. sapidus* in their diet. Research on the Chinese mitten crab *Eriocheir sinensis* H. Milne Edwards, 1853 in the Elbe River in Germany reported high nutritional value and very low concentrations of heavy metals in edible tissues (Nędzarek and Czerniejewski 2021). Similarly, Kampouris et al. (2021), evaluated the composition of the tail muscle of another invasive decapod, *Penaeus aztecus* Ives, 1891. Their findings suggested that this prawn species could serve as a substitute for the endangered spiny lobster *Palinurus elephas* (Fabricius, 1787) due to their similar nutritional values, while also acting as a control measure for the invasive population. However, managing invasive species like *C. sapidus* presents complex challenges and removal efforts must be sufficient to reduce population density while avoiding unintended impacts on native species through bycatch.

## Conclusions

The results of this study show that wetlands, such as Es Grau and S’Albufereta, act as critical points for the accumulation of heavy metals, especially Hg, due to their unique environmental characteristics. These areas have sediments rich in organic matter and anaerobic conditions, which favor the conversion of inorganic mercury into MeHg by the action of microorganisms. This form of Hg, highly toxic and bioaccumulative, is transferred through the food web, increasing the levels observed in predatory species such as *C. sapidus*. The highest levels of Hg were detected in Es Grau, which may be influenced by its management as a nature reserve. This condition may favor a greater accumulation of Hg in the sediments, either due to the continuous input of organic matter that increases its methylation or due to less human interference that could disperse the pollutants. However, S’Albufereta, also a wetland, stands out for its higher levels of Cd and Pb. These differences could be attributed to local factors such as hydrodynamics, sediment characteristics or the historical influence of point sources of pollution.

A crucial aspect of interpreting these results is the non-homogeneous distribution of the specimens analyzed by sex and life stage. In S’Albufereta, all the specimens analyzed were juveniles and females, a fact that may significantly influence the levels of metals detected. Juveniles tend to have higher assimilation of pollutants due to their metabolic rate, while physiological differences associated with sex may also affect bioaccumulation. This distribution contrasts with other locations, where most of the specimens analyzed were adults. The exclusive sampling of juvenile females at S’Albufereta highlights a key limitation in interpreting the results. Juveniles are known to exhibit higher bioaccumulation rates due to their active metabolism and dietary differences (Nyeste et al. 2019). To better understand whether these elevated levels are site-specific or biologically driven, future studies should prioritize balanced sampling across all life stages and sexes at this and other wetlands. This lack of homogeneity highlights the need to carry out additional studies to include a more balanced sample in terms of sex and life stage in all locations. Particularly, it would be essential to analyze juveniles from other wetlands to determine whether the observed patterns are specific to S’Albufereta or if they are reproduced in other locations. This would allow us to decipher more precisely the interactions between biological and environmental factors that influence the accumulation of heavy metals in *C. sapidus*.

Concerning potential human health risks from consumption, it is important to highlight that none of the analyzed specimens exceeded the legal limits established by the European Union for human consumption (0.5 mg/kg for crustaceans). However, the high concentrations of Hg in Es Grau (0.37 μg/g) and Cd and Pb in S’Albufereta (0.18 μg/g and 0.06 μg/g, respectively) could represent a potential risk, especially if cumulative intake is considered or if similar patterns are detected in other species in the food web. The findings underscore the importance of regular monitoring, particularly in areas like Es Grau, where Hg levels approach the TWI threshold. Public health initiatives should focus on educating local communities about safe consumption patterns. Additionally, mitigation efforts, such as reducing agricultural runoff into wetlands, could help lower Cd and Pb levels at S’Albufereta. These strategies align with global recommendations for managing invasive species as both ecological challenges and economic opportunities (Mancinelli et al. 2017b; Arena et al. 2024). Overall, the results reinforce the need for future research to analyze more specifically the effect of life stage and sex of individuals on the accumulation of heavy metals. It would also be necessary to expand the studies to other locations and collect samples from juveniles to confirm whether the observed concentrations respond mainly to local factors or general biological and ecological dynamics.

## Data Availability

Data will be made available upon reasonable request.
